# Drug reaction with eosinophilia and systemic symptoms secondary to minocycline complicated by posterior reversible encephalopathy syndrome

**DOI:** 10.1016/j.jdcr.2024.06.015

**Published:** 2024-07-08

**Authors:** Cheney Jianlin Wong, Angelyn Chen Yin Lua, Yong-Kwang Tay, Sze Hwa Tan, Ratna Rajaratnam

**Affiliations:** a Department of Dermatology, Changi General Hospital, Singapore, Singapore; b Department of Laboratory Medicine, Changi General Hospital, Singapore, Singapore

**Keywords:** drug reaction with eosinophilia and systemic symptoms, minocycline, posterior reversible encephalopathy syndrome

## Introduction

Drug reaction with eosinophilia and systemic symptoms (DRESS) is an uncommon severe cutaneous adverse reaction characterized by fever, eosinophilia, and visceral organ involvement. Neurological involvement is uncommon although manifestations such as meningitis and encephalitis have been described.^1^

Posterior reversible encephalopathy syndrome (PRES) is a clinicoradiological syndrome characterized by neurological symptoms associated with bilateral white matter edema on neuroimaging. Causes of PRES include hypertensive crises, renal failure, and immunosuppressants.^2^ In this report, we describe a case of minocycline-associated DRESS complicated by PRES.

## Case report

**Fig 1:**
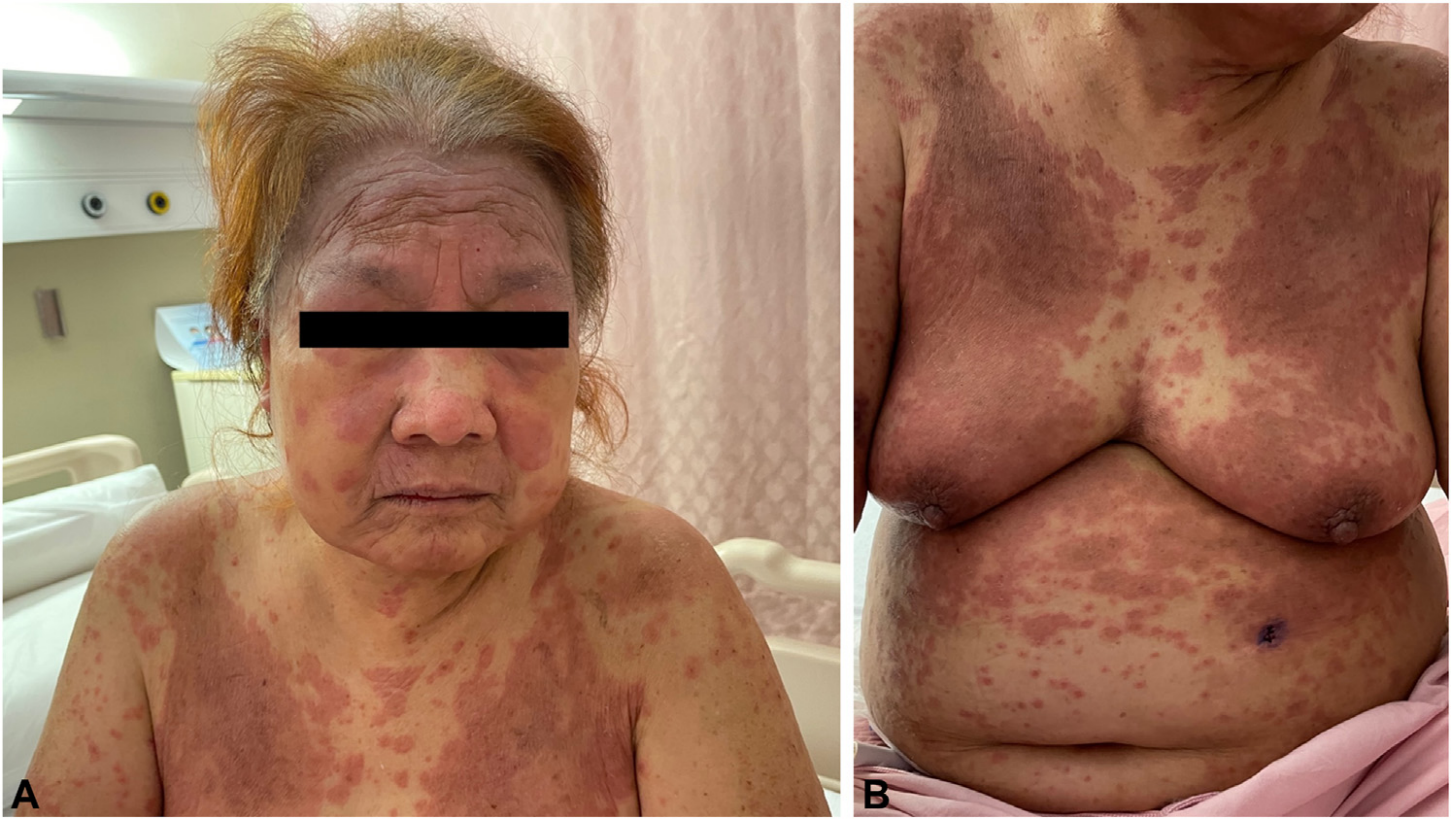
**A,** Confluent urticated plaques on face and chest. **B,** Confluent urticated plaques on anterior trunk.

A 71-year-old female presented with a fever of 38.5 °C and generalized pruritic rash of 1 month duration. Examination revealed erythematous urticated papules and plaques on her face, trunk, and limbs (Fig 1). There was mild facial edema but no lymphadenopathy. Blood tests showed acute kidney injury (serum creatinine 331 μmol/L, baseline 87 μmol/L in 2021), eosinophilia (absolute eosinophil count 5.4 × 10^9^/L), an atypical lymphocyte count of 6.0%, and elevated alkaline phosphatase (107U/L). Liver enzymes were otherwise normal.

Oral minocycline 100 mg daily had been started for chronic lichenified facial dermatitis 34 days prior to admission. She had been taking her other regular medications for at least 1 year. A diagnosis of DRESS was made based on an initial RegiSCAR score of 4 and minocycline was stopped immediately. She was commenced on oral prednisolone 30 mg daily with topical clobetasol propionate 0.05% ointment and mometasone furoate 0.1% ointment to her truncal and facial rashes, respectively.

**Fig 2:**
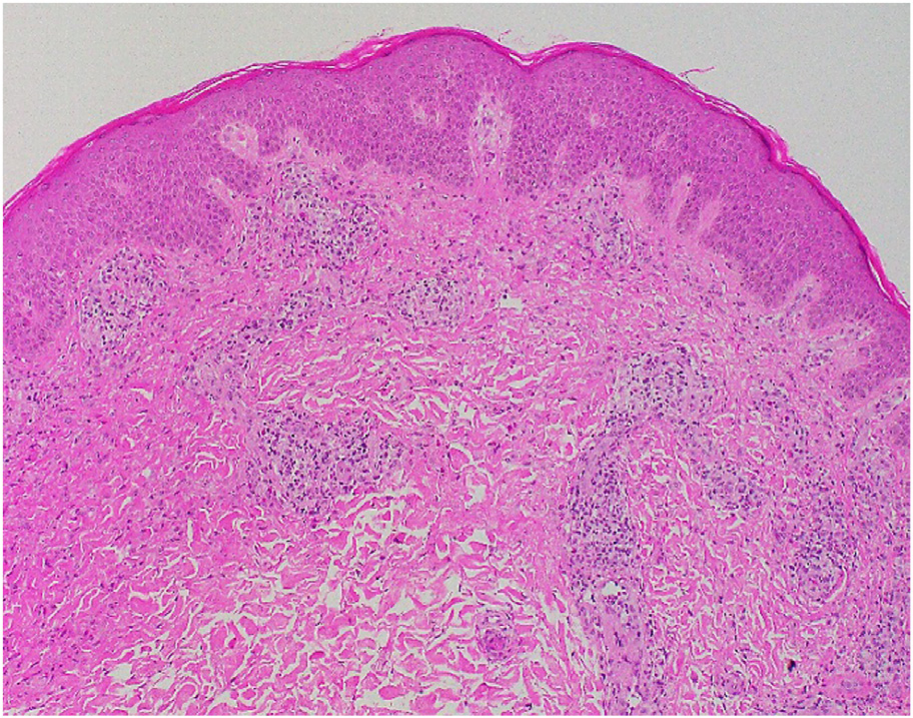
Histopathological exam with hematoxylin and eosin stain (original magnification ×100). The epidermis shows spongiotic changes while the dermis shows perivascular inflammation composed of lymphocytes, histiocytes, and some eosinophils.

Autoimmune and viral workup were unremarkable except for a slightly elevated anti-Ro antibody (2.5, normal <1.0) and a positive human herpes virus-6 polymerase chain reaction test (5970 copies/ml). Skin biopsy showed spongiotic and superficial perivascular dermatitis with eosinophilia (Fig 2). Direct immunofluorescence showed granular C3 deposits in the superficial blood vessels.

**Fig 3:**
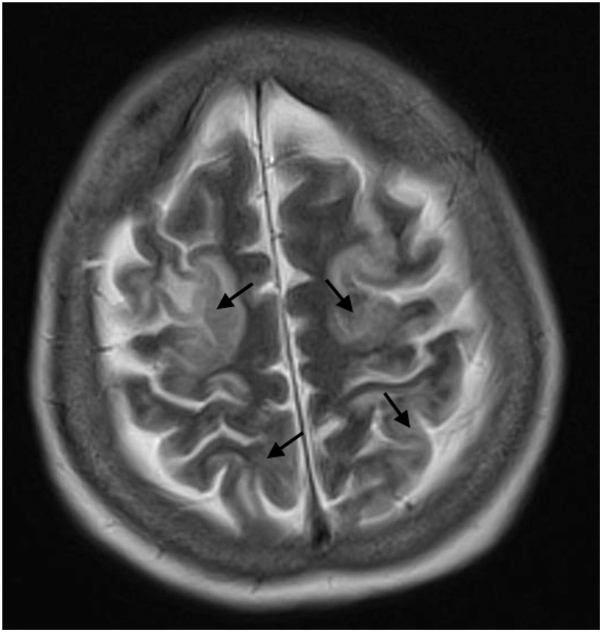
Brain magnetic resonance imaging (MRI) showing extensive T2-weighted hyperintensities involving both cortical/subcortical cerebral regions (*black arrows*).

She subsequently developed worsening renal function with oliguria and acidaemia, requiring initiation of hemodialysis 5 days after admission. Liver function tests worsened with a cholestatic picture (alkaline phosphatase 247U/L, aspartate aminotransferase 71U/L, alanine aminotransferase 24U/L and gamma-glutamyltransferase 480U/L). She then developed confusion and agitation 1 week into admission which culminated in status epilepticus requiring admission to intensive care. Her blood pressure prior to deterioration was 117/84 mmHg and this increased to 228/116 mmHg at the time of seizure activity. Brain magnetic resonance imaging (MRI) showed extensive T2-hyperintensities involving both cortical and subcortical cerebral regions (Fig 3) and a diagnosis of PRES was made.

Levetiracetam and phenytoin were started for status epilepticus. Aggressive control of blood pressure was achieved with labetalol, amlodipine, and hydralazine. Her neurological state returned to normal in 1 week and a repeat brain MRI 9 days later showed complete resolution of the widespread T2-hyperintensities. Antiepileptic medications were stopped and she had no further seizures.

Hemodialysis was stopped after 5 weeks due to renal recovery. Liver enzyme abnormalities also resolved completely. Intermittent cutaneous flares were managed with temporary increases of oral prednisolone. She remained well at her 9-month follow-up on a slow taper of oral prednisolone, currently at 10 mg daily.

## Discussion

Minocycline is a tetracycline antibiotic used in the treatment of acne vulgaris and dermatitis due to its anti-inflammatory properties.^3^ Minocycline has been associated with an increased risk of hypersensitivity reactions including DRESS.^4^ Our patient’s pre-existing dermatitis presented an initial diagnostic dilemma between disease progression and DRESS. However, the temporal relationship with drug initiation, eosinophilia, organ involvement, rash pattern, and skin biopsy findings favored DRESS.

To date, there has been 1 case report describing an association between DRESS and PRES.^5^ This involved a 68-year-old male patient with stage IV melanoma on treatment with v-raf murine sarcoma viral oncogene homolog B1/mitogen-activated protein kinase inhibitors. He developed headaches, ataxia, blurred vision, and ophthalmalgia 3 weeks after treatment initiation. Brain MRI showed new abnormal T2-hyperintensities in bilateral cerebella supporting a diagnosis of PRES. Four days later, the patient developed fever with a generalized pruritic rash over his trunk and arms. This was associated with acute kidney injury, deranged liver enzymes, and a supportive skin punch biopsy result. A diagnosis of DRESS was made.

There were some differences between our patient and the one described above. Firstly, the onset of DRESS in our patient preceded the development of PRES, whereas DRESS occurred 4 days after the onset of PRES in the other patient. Secondly, v-raf murine sarcoma viral oncogene homolog B1/mitogen-activated protein kinase inhibitors and immunotherapy are known to be independently associated with DRESS and PRES. While minocycline readily crosses the blood-brain barrier and has been reported to cause some neurological side effects such as dizziness and idiopathic intracranial hypertension,^6^ PRES is not a known adverse reaction. This suggests a likely association with DRESS and its consequential development of acute kidney injury resulting in the symptoms and signs characteristic of PRES.

In conclusion, this case highlights PRES as a possible neurological sequela of DRESS complicated by acute kidney injury. A brain MRI should be considered for patients who develop neurological symptoms in this setting so as to exclude PRES and prevent progression to permanent neurological impairment or death.

## Conflicts of interest

None disclosed.
